# Chemoenzymatic Total Synthesis of (+)-10-Keto-Oxycodone from Phenethyl Acetate †

**DOI:** 10.3390/molecules24193477

**Published:** 2019-09-25

**Authors:** Mary Ann Endoma-Arias, Helen Dela Paz, Tomas Hudlicky

**Affiliations:** Chemistry Department and Centre for Biotechnology, Brock University, 1812 Sir Isaac Brock Way St. Catharines, ON L2S 3A1, Canada; marias@brocku.ca (M.A.E.-A.); helenendomadelapaz@gmail.com (H.D.P.)

**Keywords:** enzymatic dihydroxylation, total synthesis, 10-keto-oxycodone, CAN-mediated hydroxyazidation, aminohydroxylation, pinacol-type coupling

## Abstract

The total synthesis of (+)-10-keto-oxycodone was attained from phenethyl acetate in a stereoselective manner. Absolute stereochemistry was established via enzymatic dihydroxylation of phenethyl acetate with the recombinant strain JM109 (pDTG601A) that furnished the corresponding *cis*-cyclohexadienediol whose configuration corresponds to the absolute stereochemistry of the ring C of (+)-10-keto-oxycodone. Intramolecular Heck reaction was utilized to establish the quaternary carbon at C-13, along with the dibenzodihydrofuran functionality. The C-14 hydroxyl and C-10 ketone were installed via SmI_2_-mediated radical cyclization, and oxidation of a benzylic alcohol (obtained from an intermediate nitrate azide), respectively. The synthesis of (+)-10-keto-oxycodone was completed in a total of 14 operations (21 steps) and an overall yield of ~2%. Experimental and spectral data are provided for key intermediates and new compounds.

## 1. Introduction

**Figure 1:**
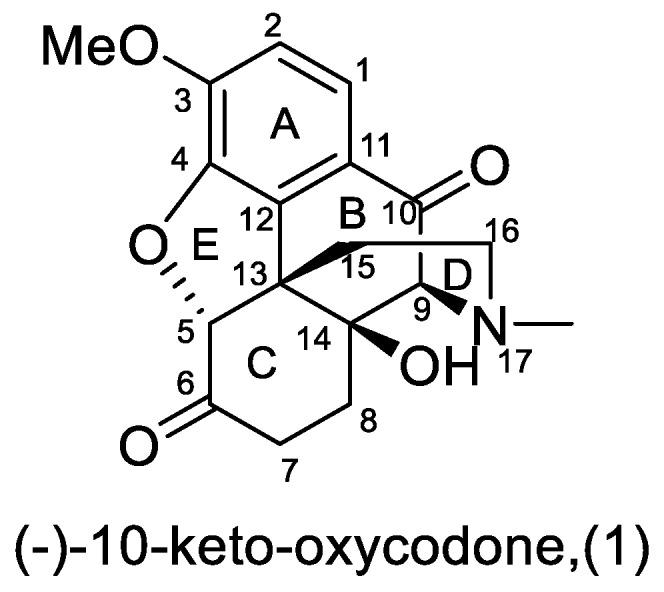
Structure of (−)-10-keto-oxycodone (natural series enantiomer, with morphine numbering system shown).

Interest in the preparation of 10-keto opiates and related derivatives such as the 10-hydroxy-morphinans was aimed at pursuing κ-selective analgesics [1]. Of the three types of opioid receptors (µ, δ, κ), the κ-opioid receptor has been especially interesting because its activation produces analgesia with minimal physical dependence [2]. A highly selective κ-opioid agonist may provide a useful analgesic free from abusive potential [3]. Among the efforts directed toward the preparation of the afore-mentioned 10-keto opiates are those aimed at the preparation of 10-keto, 10α-, 10β-hydroxy-TRK-820 [4], 10-keto-naltrexone, 10-keto-oxymorphone, 10-keto-oxycodone (Figure 1) [5], 10-keto-naloxone, and 10-keto-naloxone 3-methyl ether [6] from the corresponding opiates. These preparations are all conducted from natural morphinans by semi-syntheses. Furthermore, the above 10-keto opiates were also identified as purported intermediates in the decomposition of the parent opiates upon storage [6]. As part of our continuing program in the preparation of morphine alkaloids and related compounds [7,8], we recently had the opportunity to access a suitable intermediate that could potentially be converted to *ent*-10-keto-oxycodone.

The published methods for the preparation of 10-keto-oxycodone and 10-hydroxy-oxycodone involve the direct oxidation of oxycodone with chromium [5,9,10], or selenium dioxide [11] reagents. Recently, the use of ceric ammonium nitrate (CAN) for such an oxidation led exclusively to the 10-hydroxy product, further oxidized to the C-10-ketone with the use of stronger oxidants such as Dess–Martin periodinane [9,10]. Oxycodone, on the other hand, is prepared in two-steps from thebaine by the oxidation of the diene moiety with a peroxyacid to an enone and subsequent hydrogenation [12,13,14,15,16]. The amount of available thebaine, itself a minor constituent of opium, limits the production of oxycodone. Recently, thebaine and oripavine have become available from genetically modified poppies that produce much higher percentages of these alkaloids [17,18,19,20], now supplied by Tasmanian Alkaloids, Inc. [21]. Since the milestone synthesis of morphine by Gates [22] in 1952, there have been more than 30 total syntheses of morphine and related alkaloids and the academic pursuit continues unabated [23,24,25,26,27]. None of the reported syntheses are practical, however, for an industrial scale production. Even the most efficient academic synthesis, published by Rice [28], may not be amenable for scale-up in the industrial preparation of morphinans. Although the development of a truly practical total synthesis of any morphinan or an opiate-derived agent on a commercial scale seems to be not feasible in the near future, we have attempted to develop methods for the synthesis of oxycodone and derivatives from readily available starting materials. A de novo preparation of oxycodone and related medicinal opiate-derived agents for medicinal use could alleviate the negative impacts of any future unfavorable events that may limit the supply of natural sources—such as climate or political changes in the opium-producing areas.

## 2. Results and Discussion

**Scheme 1:**
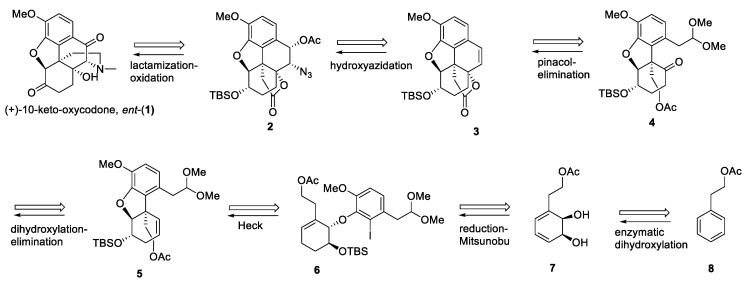
Retrosynthetic analysis of the route to *ent*-10-keto-oxycodone.

Scheme 1 outlines our retrosynthesis of (+)-10-keto-oxycodone. Disconnection of ring D leads to highly functionalized lactone **2**. In the forward sense, Fukuyama’s lactamization approach to oxycodone [29] can be utilized to construct the C-9 stereogenic center. The amino group required for the cyclization reaction is derived from hydroxyazidation of alkene lactone **3** [30,31], which is envisioned to be prepared from the keto acetal **4** following a deprotection of acetal and a SmI_2_-mediated pinacol-type coupling reaction. The key intermediate **4** can be obtained from alkene **5** via dihydroxylation followed by selective mesylation of the less hindered hydroxyl group and the elimination of the mesylate to reveal the ketone functionality in **4**. Alkene **5** is available in two steps from alcohol **7** via a sequence of steps that involves a Mitsunobu coupling with an iodo phenol acetal to furnish aryl ether **6**, followed by an intramolecular Heck reaction. The absolute stereochemistry in **7** is incorporated via microbial dihydroxylation of phenethyl acetate **8** in the whole-cell fermentation with toluene dioxygenase, overexpressed in *E. coli* JM109(pDTG601A) [32]. The enzymatically derived arene *cis*-dihydrodiols such as **7** have found widespread use in enantioselective synthesis of natural products [33,34,35,36,37,38,39,40,41,42,43,44,45,46,47,48,49,50,51].

**Scheme 2:**
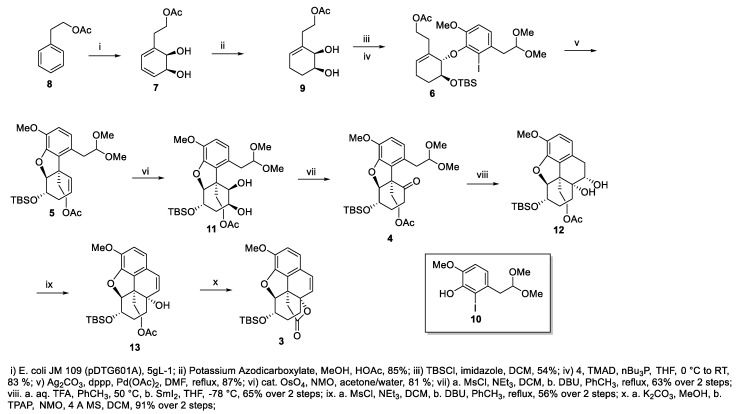
Synthesis of lactone **3**.

As previously described in our earlier publications on the preparation of *ent*-oxycodone [30,31], the synthesis began with the microbial dihydroxylation of phenethyl acetate **8** (Scheme 2) via a whole cell fermentation with *E. coli* JM109 (pDTG601A) to afford the known intermediate cyclohexadiene diol **7** (obtained in 5gL^−1^ yield) [52,53], which was subjected to a selective reduction of the more accessible alkene to afford the known diol **9** (85% yield) [54,55]. The distal, less hindered, hydroxyl in diol **9** was protected with *tert*-butyl dimethylsilyl chloride and the free allylic alcohol was then subjected to a Mitsunobu reaction with iodo phenol **10** [56], derived from isovanillin, to furnish the coupled product ether **6** (45% yield over two steps). A subsequent intramolecular Heck reaction of **6** resulted in the formation of olefin **5** (87% yield) whose osmium tetroxide-catalyzed dihydroxylation led to diol **11** (81% yield). This compound possesses the features of the three (ACE) rings of 10-keto-oxycodone. The diol functionality was converted to ketone **4** via mesylation of the less hindered hydroxyl group followed by elimination with excess 1,8-diazabicyclo[5.4.0]undec-7-ene (DBU). The mechanism may also be viewed formally as a 1,2-hydride shift. [57] of the resulting mesylate (63% yield over two steps). With the attainment of **4**, deprotection of the acetal with aqueous TFA followed by a pinacol-type coupling of the intermediate keto aldehyde using SmI_2_ afforded diol **12**, tentatively assigned as the *cis* isomer (65% yield over two steps) [58]. Mesylation of the less hindered hydroxyl group of diol **12** followed by treatment with excess DBU resulted in the formation of styrene alcohol **13** (56% yield over two steps). Hydrolysis of the acetate followed by oxidation with tetrapropylammonium perruthenate (TPAP) in the presence of *N*-methylmorpholine *N*-oxide (NMO) resulted in the formation of the key intermediate lactone **3** (91% over two steps).

**Scheme 3:**
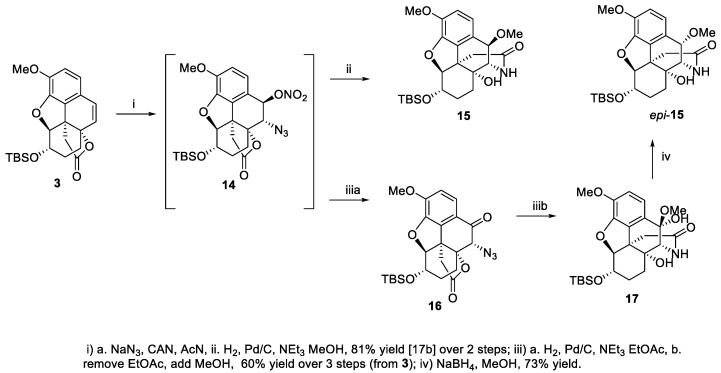
Initial studies on hydroxy lactam formation.

Based on our earlier work on a similar CAN-mediated functionalization of styrene to deliver *ent*-oxycodone [31], it was envisioned that the lactone **3** could be readily converted to a hydroxy lactam en route to *ent*-10-keto-oxycodone (Scheme 3). It was previously reported [31] that hydrogenation of azido nitrate **14** intermediate in methanol as solvent resulted in the formation benzylic methoxy ether lactam **15**. Therefore, it seemed logical that switching to non-nucleophilic solvent (e.g., EtOAc, THF) would afford the benzylic alcohol instead. To our surprise and disappointment, the reduction of azide functionality did not occur and it appears that the benzylic alcohol derived by hydrogenation of the nitrate was slowly oxidized to the ketone **16** on exposure to air during workup and further manipulation [59]. To achieve the desired reduction of the azide to amine and subsequent lactamization, the solvent had to be changed to methanol. The desired lactam formation did occur, but the product was the hemiketal **17**. This route was not followed through as this would necessitate deprotecting the hemiketal to the ketone prior to reduction with borane reagent. The hemiketal, when subjected to reduction with sodium borohydride, resulted in the formation of an ether, tentatively assigned as the C-10 epimeric benzylic methoxy ether (*epi*-**15**).

**Scheme 4:**
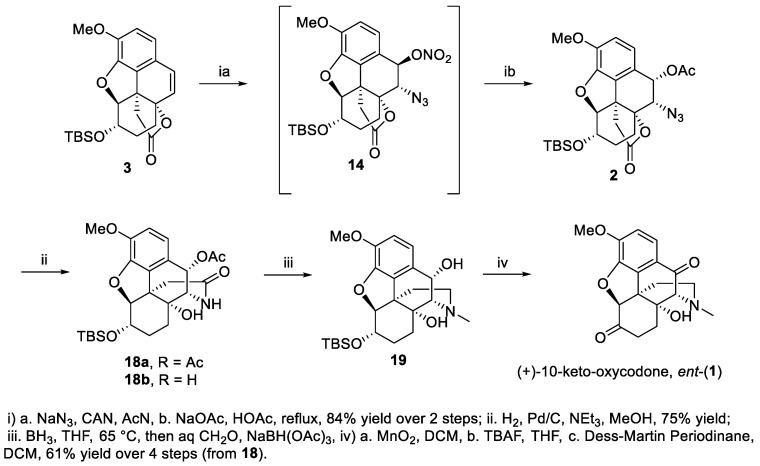
Completion of the synthesis of *ent*-10-keto-oxydocone via lactam **18**.

To address the above issue of the premature oxidation of C-10, the intermediate azido nitrate **14** was subjected to nucleophilic substitution with acetate to afford an intermediate acetate azide **2** (Scheme 4), with the acetate serving as a “protecting group” against premature oxidation of C-10. An overall inversion of stereochemistry of benzylic (C-10) carbon was tentatively assigned as in **2**, in contrast with the formation of **15** (retention). It is believed that **15** was formed from an intermediate benzylic alcohol upon hydrogenolysis of the nitrate ester followed by lactamization and then nucleophilic displacement of OH by methoxy group. The stereochemical properties of the intermediate benzylic alcohol lactam favored a beta face attack by methanol as nucleophile. The facile displacement of OH in the benzylic position was facilitated by the highly activating methoxy group at the *para* position. For the formation of *epi*-**15**, on the other hand, the hydride served as the nucleophile and thus in the product the methoxy group ended up on the alpha face. The assignment of **15** and *epi*-**15** as beta and alpha epimers was based on the coupling constants (*J*_9-10_) between the H-10 (benzylic C-H) and H-9 on similar systems [4,60]. For compounds similar to **15**, *J* values are approximately 5.0 Hz and for *epi*-**15**, *J* values are around 0 Hz. The acetate azide **2** was hydrogenated to the amine, which underwent a spontaneous lactam formation to afford the acetate lactam **18** (63% yield over three steps). Reduction of the lactam **18** with borane followed by reductive amination with formaldehyde converted the lactam to the *N*-methyl amine **19**. Because of the sensitivity (to basic and acidic reagents needed for the deprotection of C-6 hydroxyl) of the benzylic alcohol functionality in **19**, it was first oxidized with manganese dioxide to the corresponding ketone to increase stability. It has been suggested by one of the referees that the benzylic alcohol **19** may be subject to a Grob-type fragmentation under basic conditions. Deprotection of the TBS group using TBAF and oxidation of the C-6 hydroxyl afforded *ent*-10-keto-oxycodone (61% yield over four steps).

In conclusion, a relatively short chemoenzymatic total synthesis of *ent*-10-keto-oxycodone has been accomplished in 14 operations from phenethyl acetate. To the best of our knowledge this accomplishment constitutes the first synthesis of *ent*-10-keto-oxycodone.

## 3. Experimental Section

### 3.1. General Methods

Inoculum was obtained from viable cells stored −78 °C in cryovials. They were grown in suitable media as previously described [28]. The substrate was fed in 5 mL increments over the course of ~3 h with metabolites being harvested in the usual manner. All non-aqueous reactions were conducted in an argon atmosphere using standard Schlenk techniques for the exclusion of moisture and air. Methylene chloride was distilled from calcium hydride, THF and toluene were dried over sodium/benzophenone. Analytical thin layer chromatography was performed on Silicycle 60 A° 250 mm TLC plates with F-254 indicator. Flash column chromatography was performed using Kieselgel 60 (230–400 mesh). Melting points were recorded on a Hoover Unimelt apparatus (Hoover, VA, USA) and are uncorrected. IR spectra were obtained on a Perkin-Elmer One FT-IR spectrometer (PerkinElmer, Waltham, MA, USA) Optical rotation was measured on a Perkin-Elmer 341 polarimeter (PerkinElmer, Waltham, MA, USA) at a wavelength of 589 nm. ^1^H and ^13^C spectra were recorded on a 300 MHz and 400 MHz Bruker spectrometer (Bruker, Billerica, MA, USA). All chemical shifts are referenced to TMS or residual nondeuterated solvent. Data of proton spectra are reported as follows: chemical shift in ppm (multiplicity: singlet (s), doublet (d), triplet (t), quartet(q) and multiplet (m)), coupling constants [Hz], integration). Carbon spectra were recorded with complete proton decoupling and the chemical shifts are reported in ppm (δ) relative to solvent resonance as internal standard. Mass spectra and high resolution mass spectra were performed by the Analytical Division at Brock University. The ^1^H and ^13^C NMR spectra of some compounds are in the Supplementary Materials.

### 3.2. (4bR,5S,6S,8aR,9S)-9-azido-6-((tert-butyldimethylsilyl)oxy)-4,5-epoxy3-methoxy-10-oxo-5,6,7,8,9,10-hexahydro-8a,4b-(epoxyethano)phenanthren-12-one (**16**)

To a stirred solution of lactone 3 (140 mg, 0.34 mmol) in AcN (5 mL) at 0 °C was added NaN_3_ (140 mg, 2.15 mmol) followed by CAN (616 mg, 1.12 mmol). After 30 min, the reaction mixture was diluted with Et_2_O/satd. NaHCO_3_ (30 mL/15 mL). The layers were separated and the aqueous layer was further extracted with Et_2_O (2 × 20 mL). The organic layers were combined, dried over MgSO_4_, filtered and concentrated to afford a residue that was used as crude in the next step. To a stirred solution of crude azido nitrate **14** (from previous step) in EtOAc (4.0 mL) was added NEt_3_ (200 µL, 1.0 mmol) and Pd/C (10 mg, 0.004 mmol). The reaction mixture was evacuated and refilled (3×) with hydrogen gas and was allowed to stir under an atmosphere of hydrogen for 12 h. (A small aliquot was taken, filtered through a plug of Celite and concentrated to afford a solid whose ^1^H NMR spectrum was obtained.) It was taken in its crude form to the next step.

**16**: ^1^H NMR (300 MHz, CDCl_3_) δ 7.63 (d, *J* = 8.4 Hz, 1H), 7.02 (d, *J* = 8.4 Hz, 1H), 4.45 (d, *J* = 7.8 Hz, 1H), 4.09 (s, 1H), 4.02 (s, 3H), 3.24–3.35 (m, 1H), 3.16 (d, *J* = 17.7 Hz, 1H), 3.01 (d, *J* = 17.7 Hz, 1H), 2.20–2.36 (m, 1H), 1.65–1.85 (m, 3H), 0.89 (s, 9H), 0.09 (s, 3H), 0.02 (s, 3H) ppm.

### 3.3. (5R,5aR,8S,8aS,13bR,14R)-8-((tert-butyldimethylsilyl)oxy)-5a,14-dihydroxy-10,14-dimethoxy-1,2,5,5a,6,7,8,8a-octahydro-5,13-methanobenzo[2,3]benzofuro[4,3a-c]azepin-3(4H)-one (**17**)

The solvent (EtOAc) of the reaction from the previous step was exchanged (for MeOH) as follows: EtOAc was removed via rotary evaporation and the resulting residue was diluted with MeOH (4.0 mL). The reaction mixture was evacuated and refilled (3×) with hydrogen gas and was allowed to stir under an atmosphere of hydrogen for another 12 h. The catalyst was separated by filtration through Celite. The Celite was washed with MeOH (2 × 15 mL). The filtrate and washings were combined and evaporated to dryness to afford a crude solid residue that was further purified by chromatography on silica gel using DCM/MeOH (9:1) as eluent to afford the hemiketal lactam product **17** as a white solid (90 mg, 59.8% yield).

**17**: ^1^H NMR (300 MHz, CDCl_3_) δ 7.47 (d, *J* = 8.4 Hz, 1H), 6.94 (d, *J* = 8.4 Hz, 1H), 6.73 (s, 1H), 4.55 (d, *J* = 6.3 Hz, 1H), 3.99 (s, 3H), 3.84 (s, 3H), 3.35–3.45 (m, 1H), 3.17 (d, *J* = 17.7 Hz, 1H), 2.80–2.90 (m, 1H), 2.59 (d, *J* = 17.7 Hz, 1H), 1.85–1.98 (m, 1H), 1.48–1.56 (m, 1H), 1.40–1.45 (m, 2H), 0.91 (s, 9H), 0.12 (s, 3H), 0.02 (s, 3H) ppm; ^13^C NMR (75 MHz, CDCl_3_) δ 189.6, 170.2, 150.8, 144.3, 137.3, 121.6, 120.2, 115.6, 95.7, 89.0, 73.7, 72.2, 56.9, 53.6, 48.5, 38.7, 27.3, 26.2, 25.7, 18.0, −4.8, −5.1 ppm; HRMS (CI+) calcd for C_24_H_34_O_7_NSi [M − H] 476.2099 found 476.2090.

### 3.4. (5S,5aR,8S,8aS,13bR,14S)-8-((tert-butyldimethylsilyl)oxy)-5a-hydroxy-10,14-dimethoxy-1,2,5,5a,6,7,8,8a-octahydro-5,13-methanobenzo[2,3]benzofuro[4,3a-c]azepin-3(4H)-one (epi-**15**)

To as stirred solution of hemiketal **17** (10 mg, 0.02 mmol) in MeOH (1.0 mL) at 0 °C was added NaBH_4_ (10 mg, 0.26 mmol). The reaction mixture was stirred at this temperature of 15 min, then it was diluted with acetone (1.0 mL), water (1.0 mL), and Et_2_O (5 mL). The layers were separated and the aqueous phase was further extracted with Et_2_O (2 × 5 mL). The combined organic extracts were washed with brine, dried over anhydrous MgSO_4_, filtered and concentrated to afford a crude solid product (7 mg, 72.5% yield). Comparison of ^1^HNMR data for **15** and *epi*-**15** clearly indicated their relationship as epimeric compounds.

*epi*-**15**: ^1^H NMR (300 MHz, CDCl_3_) δ 6.95 (d, *J* = 8.4 Hz, 1H), 6.88(d, *J* = 8.4 Hz, 1H), 6.62 (br s, 1H), 4.88 (s, 1H), 4.43 (d, *J* = 6.6 Hz, 1H), 3.89 (s, 3H), 3.43–3.49 (m, 1H), 2.97 (d, *J* = 17.4 Hz, 1H), 2.43 (d, *J* = 17.4 Hz, 1H), 1.74–2.04 (m, 1H), 1.52–1.65 (m, 3H), 0.90 (s, 9H), 0.10 (s, 3H), 0.00 (s, 3H) ppm.

### 3.5. (4bR,5S,6S,8aR,9S,10R)-9-azido-6-((tert-butyldimethylsilyl)oxy)-4,5-epoxy3-methoxy-12-oxo-5,6,7,8,9,10-hexahydro-8a,4b-(epoxyethano)phenanthren-10-yl nitrate (**14**)

To a stirred solution of lactone 3 [17b] (50 mg, 0.12 mmol) in AcN (5 mL) at 0 °C was added NaN_3_ (50 mg, 0.77 mmol) followed by CAN (220 mg, 0.40 mmol). After 30 min, the reaction mixture was diluted with Et_2_O/satd. NaHCO_3_ (10 mL/5 mL). The layers were separated and the aqueous layer was further extracted with Et_2_O (2 × 10 mL). The organic layers were combined, dried over MgSO4, filtered and concentrated to afford a residue that was used as crude in the next step. A small sample of azido nitrate **14** was subjected to purification by preparative TLC (SiO_2_ gel, 2:1 hex/EtOAc as eluent) for characterization purposes. It was found to have limited stability.

**14**: ${\left[\alpha \right]}_{D}^{20}$ = −35.8 (c = 0.6, CH_2_Cl_2_); R_f_ = 0.44 (4:1; hexanes/EtOAc); IR (film) *v* 3434, 2955, 2929, 2855, 2116, 1796, 1645, 1508, 1440, 1274, 837 cm^−1^; ^1^H NMR (300 MHz, CDCl_3_) δ 6.99 (d, *J* = 8.4 Hz, 1H), 6.96 (d, *J* = 8.4 Hz, 1H), 6.30 (d, *J* = 1.8 Hz, 1H), 4.40 (d, *J* = 7.8 Hz, 1H), 4.10 (d, *J* = 1.8 Hz, 1H), 3.95 (s, 3H), 3.25–3.29 (m, 1H), 3.04 (d, *J* = 17.7 Hz, 1H), 2.94 (d, *J* = 17.7 Hz, 1H), 2.13–2.17 (m, 1H), 1.68–1.77 (m, 3H), 0.89 (s, 9H), 0.10 (s, 3H), 0.00 (s, 3H) ppm; ^13^C NMR (75 MHz, CDCl_3_) δ 173.6, 151.8, 146.2, 128.9, 122.6, 116.5, 115.3, 99.4, 82.9, 80.5, 69.8, 67.5, 56.8, 49.6, 43.4, 29.7, 28.7, 26.7, 25.7, 18.0, −4.8, −5.2 ppm; MS (EI) *m*/*z* (%) 416(15), 386(60), 358(100), 328(30), 288(75), 272(45); HRMS (EI) calcd for C_19_H_21_O_8_N_4_Si [M − C_4_H_9_-H_2_O], 461.1123 found 461.1118.

### 3.6. (4bR,5S,6S,8aR,9S,10S)-9-azido-6-((tert-butyldimethylsilyl)oxy)4,5-epoxy-3-methoxy-12-oxo-5,6,7,8,9,10-hexahydro-8a,4b-(epoxyethano)phenanthren-10-yl acetate (**2**)

To the crude azido nitrate **14** (obtained from the previous step) dissolved in HOAc (1.5 mL) was added NaOAc (150 mg, 1.83 mmol) and the reaction mixture was heated to 100 °C for 2 h. After cooling to room temperature, the mixture was carefully diluted with satd NaHCO_3_/EtOAc (5 mL/5 mL). The layers were separated and the aqueous phase was further extracted with EtOAc (2 × 5 mL). The combined organic extracts were washed with brine, dried over anhydrous MgSO_4_, filtered and concentrated to afford a residue that was subjected to chromatography on silica gel using hexanes/EtOAc (2:1) as eluent to afford the product **2** as an oil (52 mg, 84% yield over two steps).

**2**: ${\left[\alpha \right]}_{D}^{20}$ = −2.4 (c = 1.0, CH_2_Cl_2_); R_f_ = 0.50 (2:1; hexanes/EtOAc); IR (film) *v* 2954, 2928, 2855, 2113, 1796, 1747, 1632, 1507, 1441, 1264, 1125, 838, 738 cm^−1^; ^1^H NMR (300 MHz, CDCl_3_) δ 6.92 (d, *J* = 8.4 Hz, 1H), 6.87 (d, *J* = 8.4 Hz, 1H), 6.20 (d, *J* = 2.7 Hz, 1H), 4.42 (d, *J* = 7.8 Hz, 1H), 3.94 (s, 3H), 3.90 (d, *J* = 2.7 Hz, 1H), 3.28–3.36 (m, 1H), 3.13 (d, *J* = 17.7 Hz, 1H), 3.04 (d, *J* = 17.7 Hz, 1H), 2.19–2.25 (m, 1H), 2.12 (s, 3H), 1.66–1.79 (m, 3H), 0.90 (s, 9H), 0.12 (s, 3H), 0.02 (s, 3H) ppm; ^13^C NMR (75 MHz, CDCl_3_) δ 173.9, 169.9, 145.8, 145.7, 127.8, 121.4, 120.4, 116.3, 99.3, 83.5, 71.3, 70.3, 69.1, 56.8, 49.8, 43.7, 29.7, 29.3, 26.8, 25.7, 21.1, 18.0, −4.7, −5.2 ppm; MS (EI) *m*/*z* (%) 458(80), 430(20), 398(40), 370(100), 343(40), 328(80), 300(45), 254(45), 117(90); HRMS (EI) calcd for C_21_H_24_O_7_N_3_Si [M − C_4_H_9_], 458.1378 found 458.1373.

### 3.7. (4S,4aR,7S,7aS,12bR,13S)-7-((tert-butyldimethylsilyl)oxy)-4a-hydroxy-9-methoxy-2-oxo-2,3,4,4a,5,6,7,7a-octahydro-1H-4,12-methanobenzofuro[3,2-e]isoquinolin-13-yl acetate (**18a**)

To a stirred solution of azido acetate **2** (20 mg, 0.04 mmol) in MeOH (0.5 mL) was added NEt_3_ (30 µL, 0.15 mmol) and Pd/C (2.5 mg, 0.001 mmol). The reaction mixture was evacuated and refilled (3×) with hydrogen gas and was allowed to stir under an atmosphere of hydrogen for 12 h. The catalyst was separated by filtration through Celite. The Celite was washed with MeOH (2 × 5 mL). The filtrate and washings were combined and evaporated to dryness to afford a crude solid residue that was further purified by chromatography on silica gel using DCM/MeOH (9:1) as eluent to afford the desired acetate lactam product 18a and the corresponding alcohol lactam **18b** in a ratio of 8:1 as white solids (13 mg, 75% yield). A small sample of acetate lactam was hydrolyzed to the alcohol lactam for characterization purposes.

**18a**: ${\left[\alpha \right]}_{D}^{20}$ = 48.0 (c = 0.4, CH_2_Cl_2_); m.p. 140–141 °C; R_f_ = 0.42 (9:1; CH_2_Cl_2_/MeOH); IR (film) *v* 3336, 2929, 2855, 1733, 1632, 1608, 1366, 1234, 1021, 837, 755 cm^−1^; ^1^H NMR (300 MHz, CDCl_3_) δ 6.88 (s, 2H), 6.77 (d, *J* = 4.5 Hz, 1H), 5.95 (d, *J* = 2.0 Hz, 1H), 4.49 (d, *J* = 6.0 Hz, 1H), 3.92 (s, 3H), 3.68 (dd, *J* = 4.5 Hz, 2.0 Hz, 1H), 3.50–3.53 (m, 1H), 2.90 (d, *J* = 17.7 Hz, 1H), 2.48 (d, *J* = 17.7 Hz, 1H), 2.10 (s, 3H), 1.8–1.97 (m, 2H), 1.63–1.68 (m, 1H), 1.50–1.53 (m, 1H), 0.92 (s, 9H), 0.15 (s, 3H), 0.05 (s, 3H) ppm; ^13^C NMR (75 MHz, CDCl_3_) δ 171.1, 170.5, 146.0, 144.3, 133.7, 122.1, 119.5, 115.9, 96.0, 73.1, 72.2, 69.0, 60.9, 56.8, 46.7, 39.9, 29.6, 26.3, 25.7, 21.1, 18.0, −4.7, −5.1 ppm; MS (EI+) *m*/*z* (%) 433(25), 432(80), 373(40), 372(100), 354(20), 330(20), 285(65), 274(75); HRMS (EI) calcd for C_25_H_36_O_7_NSi [M + H], 490.2256 found 490.2261.

### 3.8. (4S,4aR,7S,7aS,12bR,13S)-7-((tert-butyldimethylsilyl)oxy)-4a,13-dihydroxy-9-methoxy-4,4a,5,6,7,7a-hexahydro-1H-4,12-methanobenzofuro[3,2-e]isoquinolin-2(3H)-one (**18b**)

**18b**: ${\left[\alpha \right]}_{D}^{20}$ = 135.8 (c = 0.3, CH_2_Cl_2_); m.p. 135–137 °C; R_f_ = 0.37 (9:1; CH_2_Cl_2_/MeOH); IR (film) *v* 3391, 2954, 2929, 2856, 1644, 1634, 1440, 1258, 838 cm^−1^; ^1^H NMR (300 MHz, CDCl_3_) δ 7.73 (br s, 1H), 6.87 (d, *J* = 8.4 Hz, 1H), 6.82 (d, *J* = 8.4 Hz, 1H), 4.88 (br s, 1H), 4.44 (d, *J* = 6.0 Hz, 1H), 3.89 (s, 3H), 3.69 (s, 1H), 3.43–3.49 (m, 1H), 2.88 (d, *J* = 17.7 Hz, 1H), 2.63 (d, *J* = 17.7 Hz, 1H), 1.98–2.15 (m, 1H), 1.79–1.91 (m, 1H), 1.55–1.59 (m, 1H), 1.41–1.44 (m, 1H), 0.91 (s, 9H), 0.12 (s, 3H), 0.02 (s, 3H) ppm; ^13^C NMR (75 MHz, CDCl_3_) δ 172.7, 145.5, 144.4, 132.6, 123.2, 121.9, 115.7, 96.5, 72.4, 69.2, 66.6, 63.4, 56.7, 46.6, 39.6, 29.7, 26.5, 25.8, 18.0, −4.8, −5.1 ppm; MS (EI) *m*/*z* (%) 390(70), 372(100), 330(25), 285(95), 274(60), 259(25); HRMS (EI) calcd for C_19_H_24_O_6_NSi [M − C_4_H_9_], 390.1367 found 390.1370.

### 3.9. (4S,4aR,7S,7aS,12bR,13S)-7-((tert-butyldimethylsilyl)oxy)-9-methoxy-3-methyl-1,2,3,4,5,6,7,7a-octahydro-4aH-4,12-methanobenzofuro[3,2-e]isoquinoline-4a,13-diol (**19**)

To a stirred solution of acetate lactam **18a** (60 mg, 0.12 mmol) in THF (1.5 mL) at 0 °C was added BH_3_ in THF (6.8 mL, 6.6 mmol). After stirring at this temperature for 10 min, the mixture was heated to 65 °C for 16 h. The reaction mixture was allowed to cool to room temperature and quenched by the dropwise addition of MeOH (5 mL) and acetic acid (500 µL). To the reaction mixture was added 30% aq. CH_2_O (1.2 mL, 11.2 mmol) and NaBH(OAc)_3_ (650 mg, 3.03 mmol) at room temperature. After 1.5 h, the reaction mixture was diluted with DCM/satd. NaHCO_3_ (30 mL/30 mL). The layers were separated and the aqueous phase was further extracted with DCM (2 × 30 mL). The organic layers were combined, dried over MgSO_4_, filtered and concentrated to afford a residue that was used as crude in the next step. A small sample was taken whose ^1^H NMR spectrum was obtained. This compound has limited stability so it was taken as a crude product to the next step.

**19**: ^1^H NMR (300 MHz, CDCl_3_) δ 6.94 (d, *J* = 8.4 Hz, 1H), 6.86 (d, *J* = 8.4 Hz, 1H), 5.08 (s, 1H), 4.45 (d, *J* = 6.3 Hz, 1H), 3.92 (s, 3H), 3.43–3.51 (m, 1H), 2.86 (s, 1H), 2.55 (s, 3H), 2.50–2.60 (m, 1H), 2.17–2.25 (m, 1H), 1.97–2.05 (m, 2H), 1.59–1.67 (m, 2H), 1.4–1.55 (m, 2H), 0.91 (s, 9H), 0.13 (s, 3H), 0.03 (s, 3H) ppm.

### 3.10. ent-10-keto-oxycodone (ent-(**1**))

To the crude N-methyl amine alcohol 19 (obtained from the previous step) dissolved in DCM (1.0 mL) was added MnO_2_ (191 mg, 2.2 mmol). The suspension was stirred vigorously at room temperature for 1 day. The reaction mixture was filtered through a plug of Celite and the residue was washed with DCM (5 mL). The filtrate was concentrated by rotary evaporation and the residue was used as crude in the next step. To a stirred solution of N-methyl ketone (obtained from the previous step) in THF (1 mL) was added TBAF (250 µL, 0.25 mmol). The reaction mixture was stirred at room temperature for 5 h after which it was diluted with EtOAc/H_2_O (5 mL/5 mL). The layers were separated and the aqueous layer was further extracted with DCM (2 × 5 mL). The organic layers were combined, dried over MgSO_4_, filtered and concentrated to afford a residue that was used as crude in the next step. To the crude alcohol dissolved in DCM (3 mL) was added Dess-Martin periodinane (93.3 mg, 0.22 mmol). The reaction mixture was stirred at rt for 2 h. It was diluted with satd. Na_2_S_2_O_3_ (1 mL), followed by satd. NaHCO_3_ solution (1 mL). The layers were separated and the aqueous phase was further extracted with DCM (2 × 10 mL). The organic layers were combined, dried over MgSO_4_, filtered and concentrated to afford a residue that was chromatographed on silica gel using DCM/MeOH as eluent (9:1) to afford the product *ent*-(**1**) as a solid (24 mg, 0.07 mmol, 60.7% yield over four steps).

*ent*-(**1**): ${\left[\alpha \right]}_{D}^{20}$ = 156.8 (c = 0.3, CH_2_Cl_2_); m.p. 239–241 °C, lit. [5] m.p. 242–243 °C R_f_ = 0.57 (9:1; CH_2_Cl_2_/MeOH); IR (film) *v* 3434, 2924, 2851, 1727, 1674, 1619, 1443, 1291, 1115 cm^−1^; ^1^H NMR (300 MHz, CDCl_3_) δ 7.46 (d, *J* = 8.4 Hz, 1H), 6.88 (d, *J* = 8.4 Hz, 1H), 4.84 (br s, 1H), 4.77 (s, 1H), 4.03 (s, 3H), 3.09 (dd, *J* = 14.4, 4.8 Hz, 3.02 (s, 1H), 2.57–2.72 (m, 2H), 2.49 (s, 3H), 2.20–2.38 (m, 2H), 1.92–1.96 (m, 1H), 1.65–1.77 (m, 1H), 1.45–1.63 (m, 1H) ppm; ^13^C NMR (75 MHz, CDCl_3_) δ 207.2, 192.5, 150.2, 144.9, 136.8, 123.8, 119.5, 115.0, 89.9, 73.7, 71.2, 56.9, 52.0, 45.7, 43.3, 35.9, 31.6, 29.6 ppm; MS (EI) *m*/*z* (%) 329(100), 273(25), 244(25), 217(30), 112(10); HRMS (EI+) calcd for C_18_H_19_O_5_N, 329.1258 found 329.1258.

## Supplementary Materials

Copies of spectral data (^1^H NMR and ^13^C NMR) are provided for the following compounds: **2**, **18a**, **18b**, **14**, *ent*-(**1**).
