# Supplementary material for: Chemoenzymatic Total Synthesis of (+)-10-Keto-Oxycodone from Phenethyl Acetate
Source: Molecules. 2019 Sep 25;24(19):3477. doi: 10.3390/molecules24193477 (PMC6804203; doi:10.3390/molecules24193477)

Supporting Information

Chemoenzymatic total synthesis of (+)-10-keto-oxycodone from phenethyl acetate

Mary Ann A. Endoma-Arias, Helen E. Dela Paz and Tomas Hudlicky*

Chemistry Department and Centre for Biotechnology, Brock University, 1812 Sir Isaac Brock Way St. Catharines, Ontario, Canada L2S 3A1

thudlicky@brocku.ca

Table of Contents

NMR spectra of (4bR,5S,6S,8aR,9S,10R)-9-azido-6-((tert-butyldimethylsilyl)oxy)-4,5-epoxy3-methoxy-12-oxo-5,6,7,8,9,10-hexahydro-8a,4b-(epoxyethano)phenanthren-10-yl nitrate (**14**)……………………………………………………………………………2

NMR spectra of (4bR,5S,6S,8aR,9S,10S)-9-azido-6-((tert-butyldimethylsilyl)oxy)4,5-epoxy-3-methoxy-12-oxo-5,6,7,8,9,10-hexahydro-8a,4b-(epoxyethano)phenanthren-10-yl acetate (**2**)……………………………………………………………...…………3

NMR spectra of (4S,4aR,7S,7aS,12bR,13S)-7-((tert-butyldimethylsilyl)oxy)-4a-hydroxy-9-methoxy-2-oxo-2,3,4,4a,5,6,7,7a-octahydro-1H-4,12-methanobenzofuro[3,2-e]isoquinolin-13-yl acetate (**18a**)……………………………………………...……4

NMR spectra of (4S,4aR,7S,7aS,12bR,13S)-7-((tert-butyldimethylsilyl)oxy)-4a,13-dihydroxy-9-methoxy-4,4a,5,6,7,7a-hexahydro-1H-4,12-methanobenzofuro[3,2-e]isoquinolin-2(3H)-one (**18b**)…………………………………………………...…5

NMR spectra of *ent*-10-keto-oxycodone **(1)**…………………………………………6

^{1}HNMR (300 MHz, $CDCl_3$)

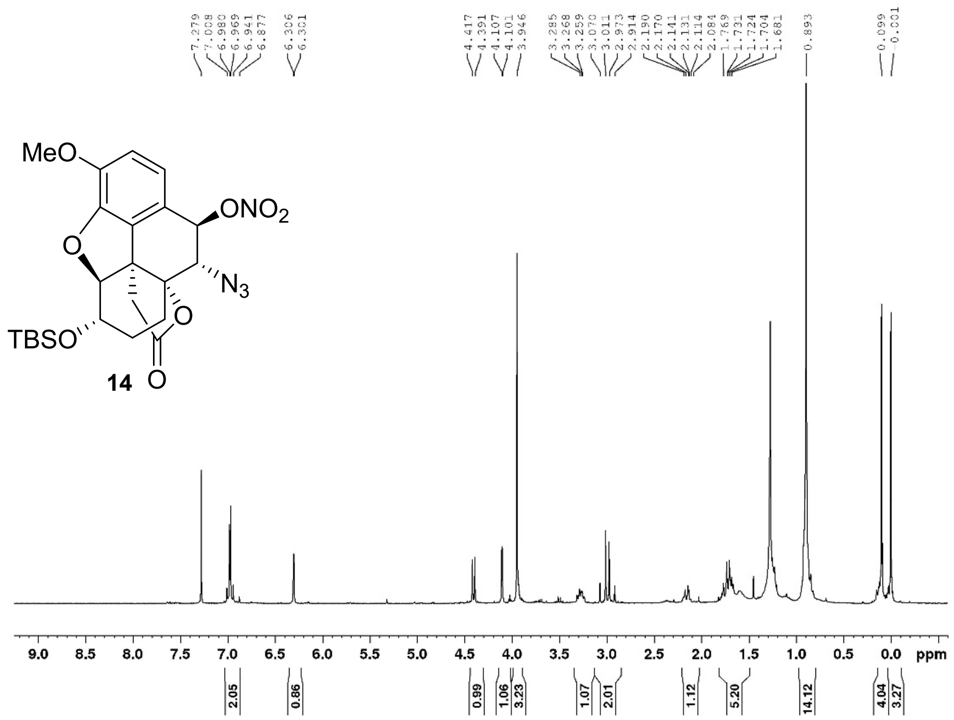

^{13}CNMR (75 MHz, $CDCl_3$)

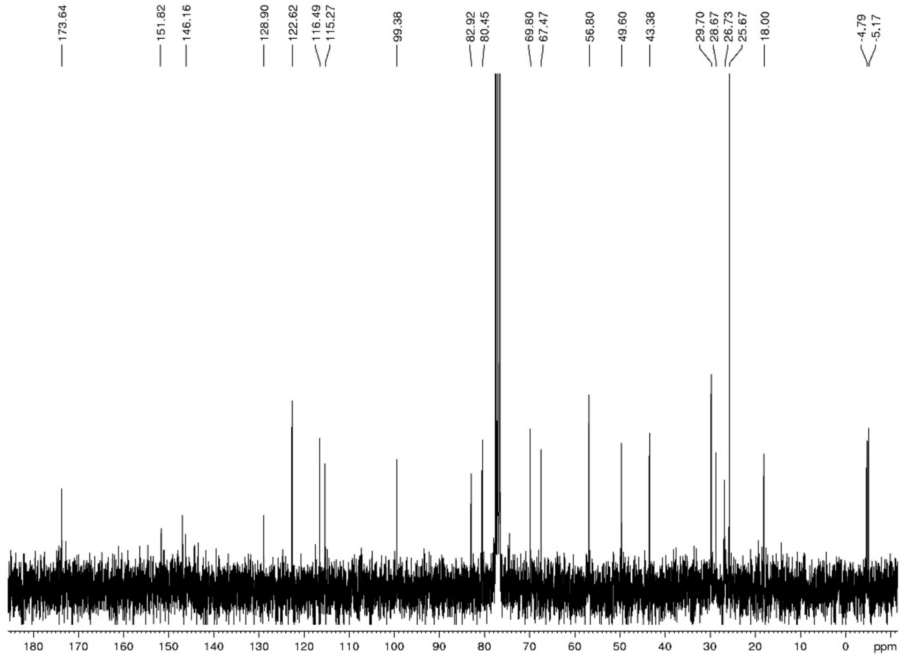

^{1}HNMR (300 MHz, $CDCl_3$)

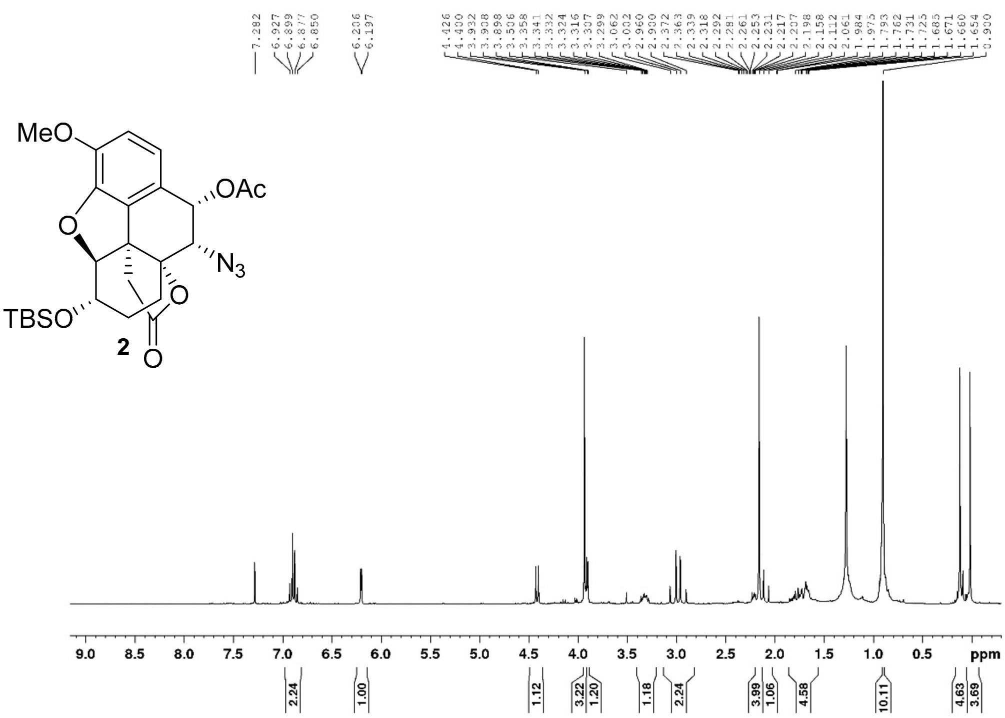

^{13}CNMR (75 MHz, $CDCl_3$)

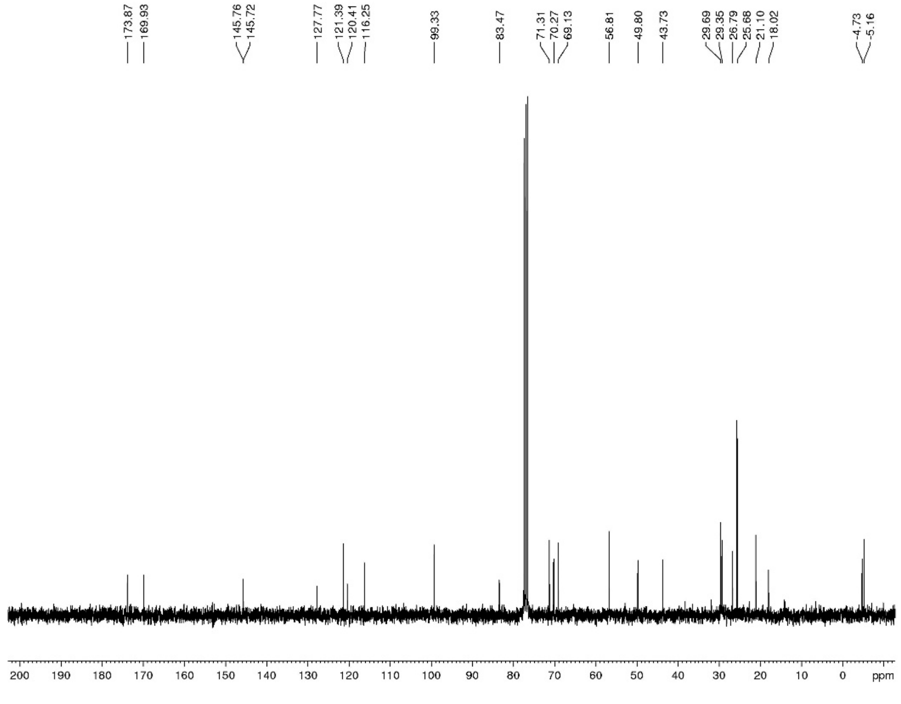

^{1}HNMR (300 MHz, $CDCl_3$)

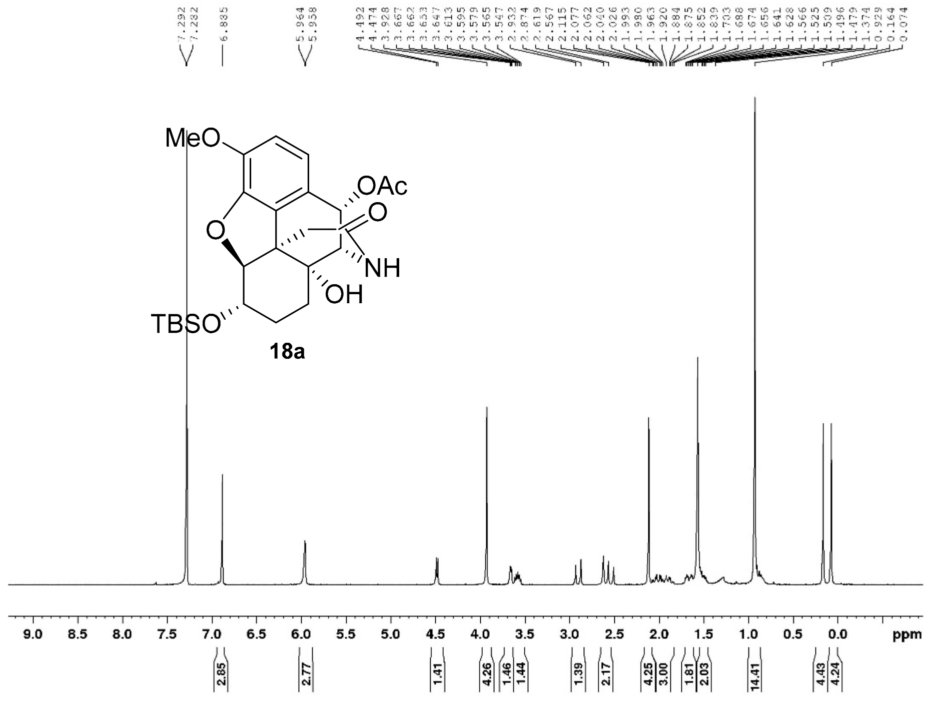

^{13}CNMR (75 MHz, $CDCl_3$)

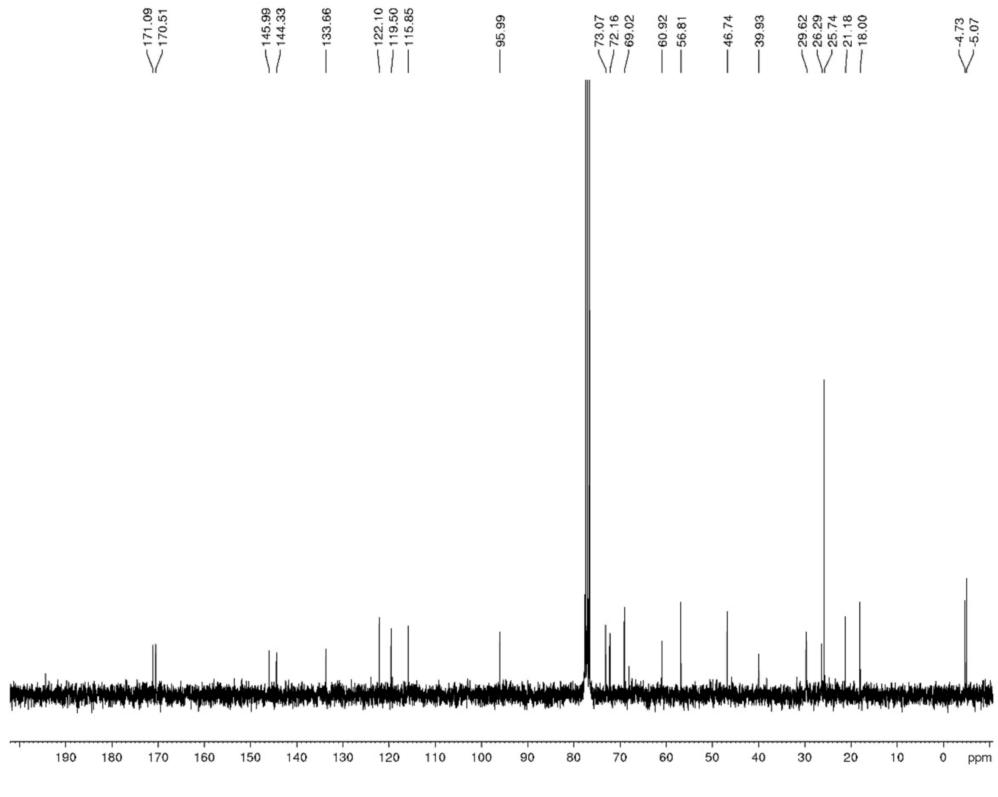

^{1}HNMR (300 MHz, $CDCl_3$)

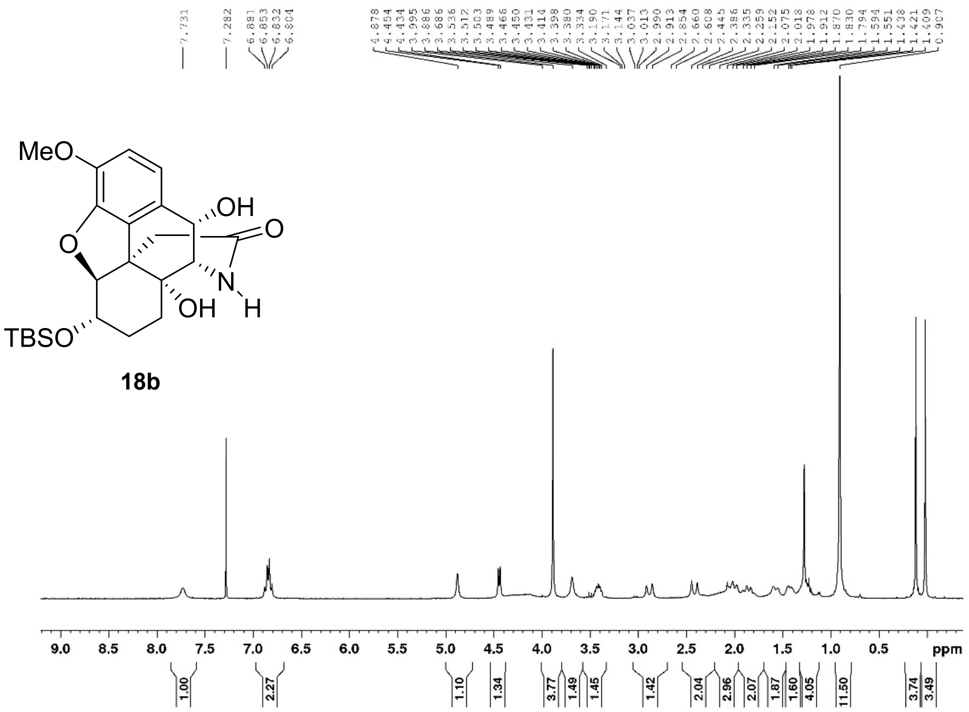

^{13}CNMR (75 MHz, $CDCl_3$)

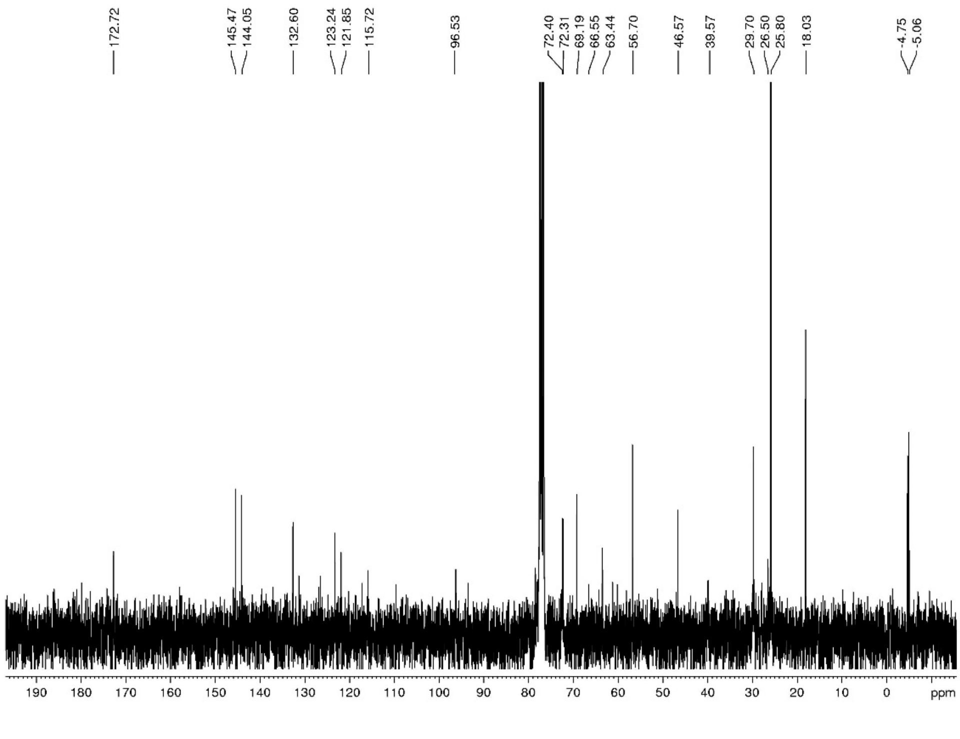

^{1}HNMR (300 MHz, $CDCl_3$)

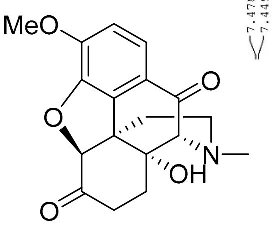

(+)-10-keto-oxycodone, *ent*-(**1**)

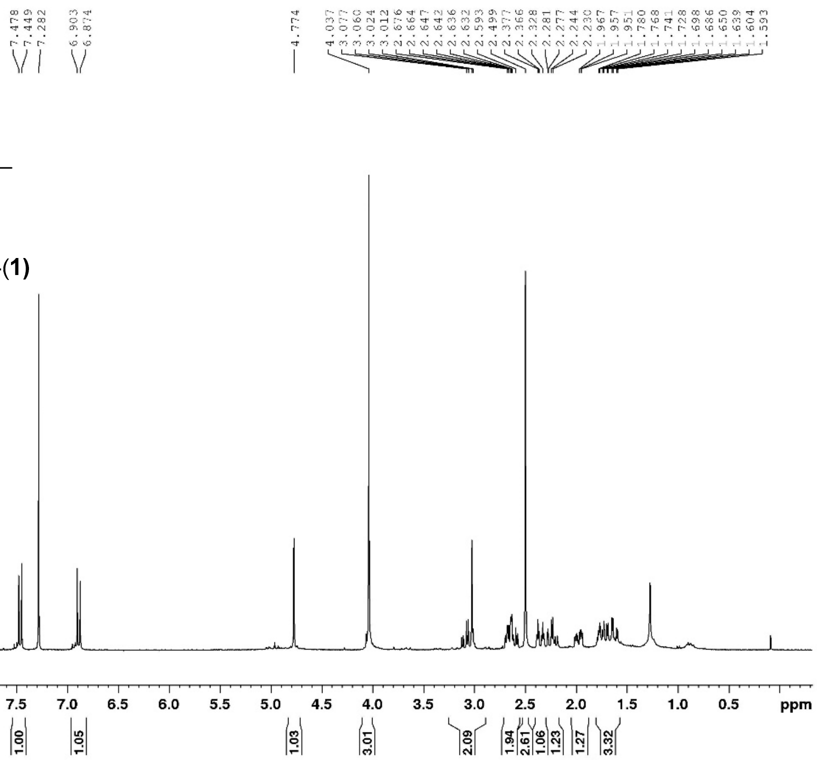

^{13}CNMR (75 MHz, $CDCl_3$)

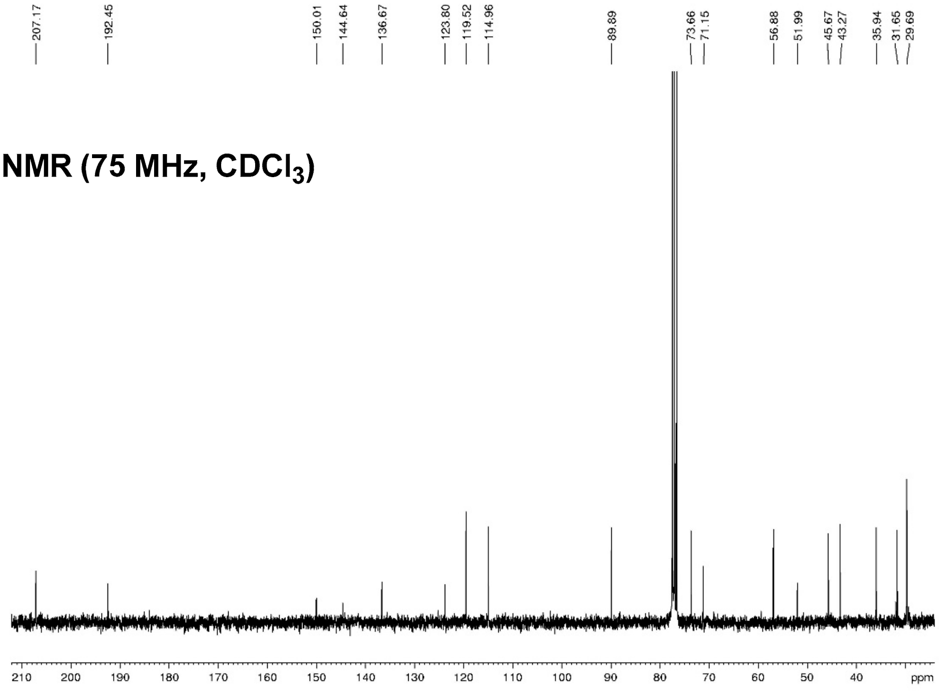

Supplement: Supplementary file 1 [file molecules-24-03477-s001.pdf]
